# Neurovascular Unit: A New Target for Treating Early Stages of Diabetic Retinopathy

**DOI:** 10.3390/pharmaceutics13081320

**Published:** 2021-08-23

**Authors:** Rafael Simó, Olga Simó-Servat, Patricia Bogdanov, Cristina Hernández

**Affiliations:** 1 Diabetes and Metabolism Research Unit, Vall d’Hebron Research Institute (VHIR), 08035 Barcelona, Spain; olga.simo@vhir.org (O.S.-S.); patricia.bogdanov@vhir.org (P.B.); cristina.hernandez@vhir.org (C.H.); 2 Centro de Investigación Biomédica en Red de Diabetes y Enfermedades Metabólicas Asociadas (CIBERDEM), Instituto de Salud Carlos III (ICSIII), 28029 Madrid, Spain

**Keywords:** diabetic retinopathy, treatment, neurovascular unit, neurodegeneration, eye drops, early stages of diabetic retinopathy, neuroprotection

## Abstract

The concept of diabetic retinopathy as a microvascular disease has evolved and is now considered a more complex diabetic complication in which neurovascular unit impairment plays an essential role and, therefore, can be considered as a main therapeutic target in the early stages of the disease. However, neurodegeneration is not always the apparent primary event in the natural story of diabetic retinopathy, and a phenotyping characterization is recommendable to identify those patients in whom neuroprotective treatment might be of benefit. In recent years, a myriad of treatments based on neuroprotection have been tested in experimental models, but more interestingly, there are drugs with a dual activity (neuroprotective and vasculotropic). In this review, the recent evidence concerning the therapeutic approaches targeting neurovascular unit impairment will be presented, along with a critical review of the scientific gaps and problems which remain to be overcome before our knowledge can be transferred to clinical practice.

## 1. Introduction

Diabetic retinopathy (DR) is the most common complication of diabetes and remains the leading cause of blindness among working-age individuals in developed countries [1,2,3]. In addition, given that diabetes is expected to increase from 464 million in 2020 to 600 million in 2035, DR will become an even more serious problem in the next few years [4]. For this reason, a multidisciplinary largescale task force integrating clinical and molecular information is urgently needed to design new effective treatments to prevent and arrest this devastating complication of diabetes. This strategy will only have a significant societal impact when the new metrics for assessing the progression of the disease [5], which allow us to shorten the duration of clinical trials, will be approved by the regulatory authorities.

Since DR remains asymptomatic until the pathology is significantly advanced, screening to detect it during the early stages is necessary [6]. However, current treatments (ex. laser photocoagulation, intravitreal injections of corticosteroids or anti-VEGF agents) target late stages of DR when vision has already been significantly affected. Therefore, during a large period of the natural history of the disease, we are not treating DR with drugs directly addressed to the retinal pathology, and the only treatment that we are offering to the patients is a tight control of the modifiable risk factors, such as blood glucose levels and hypertension. This scenario is really frustrating in the 21st century and novel and more efficient preventional and interventional strategies against early stages of DR are mandatory. In this article, the recent evidence regarding the beneficial effects of drugs acting at early stages of the disease will be provided along with a critical review of problems which have yet to be overcome.

## 2. The Neurovascular Unit

The concept of DR as a microvascular disease has evolved, in that it is now considered a more complex diabetic complication in which neurodegeneration plays a significant role [7,8,9,10]. In fact, the American Diabetes Association (ADA) in its most recent position statement defined DR as a highly tissue-specific neurovascular complication [11]. As as is the case in the brain, the neurovascular unit (NVU) in the retina refers to the functional coupling and interdependency of neurons, glia, and the highly specialized vasculature. The components of the NVU include diverse neural cell types (i.e., ganglion cells, bipolar, amacrine, and horizontal bipolar cells), glia (Müller cells, astrocytes, and microglia), and vascular cells (endothelial cells and pericytes) [12,13,14]. All these components are in intimate communication and maintain the integrity of the inner blood–retinal barrier (iBRB) whilst dynamically regulating blood flow in response to metabolic demands [15,16]. Within the NVU, some types of cells, such as glial cells, endothelial cells, and pericytes, can communicate directly through physical interactions, while cells without physical connections communicate at distance via secreted soluble ligands and/or exosomes.

Neuronal and glial-mediated neurovascular coupling is essential for the normal homeostatic function of NVU. Therefore, neurodegeneration and glial activation are primary events in the pathogenesis of DR, and have been observed to occur before overt microangiopathy in experimental models of DR and in the retina of diabetic donors [17,18,19].

Diabetes-induced retinal neurodegeneration consists of a progressive loss of retinal neurons such as ganglion cells, amacrine cells, and photoreceptors, which accounts for the early electrophysiological deficits that occur in the natural story of DR, as well as impairment in color vision (in particular blue-sensitive defects) and decreased contrast sensitivity [20,21,22,23,24,25]. In addition, it should be noted that the diabetes-induced alterations of photoreceptors and retinal pigment epithelium (RPE) also have deleterious consequences on the retinal microvasculature [26]. Furthermore, a relationship between early structural damage of neuroretina and glucose variability has been reported in type 1 diabetic patients [27].

One of the main lessons of the European Consortium for the Early Treatment of Diabetic Retinopathy (EUROCONDOR) clinical trial was that neurodegeneration (at least when assessed by multifocal electroretinography) was not identified in a significant proportion (35%) of patients with early microvascular impairment (ETDRS 20-35) [28,29]. Therefore, neurodegeneration could be the herald of DR only in a subset of subjects with diabetes. This finding points to screening retinal neurodysfunction as a critical issue for identifying those patients in whom neuroprotective treatment might be of benefit [14].

## 3. Current Treatments for Early Stages of Diabetic Retinopathy

At present, there are no drugs recognized by any Scientific Academic Society for the treatment of the early stages of DR. Therefore, only general measures, such as tight metabolic control in terms of blood glucose levels and lipids, as well as good control of blood pressure, are recommended. Nevertheless, two drugs (fenofibrate and calcium dobesilate) have been shown to be effective and safe in treating DR in several clinical trials and, therefore, merit to be commented.

### 3.1. Fenofibrate

Fenofibrate is a peroxisome proliferator-activated receptor alpha (PPARα) currently used as a hypolipidemic agent. However, two seminal prospective randomized controlled trials (the FIELD [30] and the ACCORD-Eye [31] studies) have shown that DR progression in type 2 diabetes is significantly reduced by fenofibrate with an excellent NNT. Therefore, it seems reasonable to recommend fenofibrate to prevent DR progression in patients with preexisting disease. A large randomized, double-masked, placebo-controlled clinical trial addressed to evaluate the effect of fenofibrate for the prevention of DR worsening is ongoing in the US. This study, comprising 910 participants with 4 years of follow-up, is expected to finish in April 2027 and should further enlighten the scientific community regarding the usefulness and safety of fenofibrate for treating early stages of DR (ClinicalTrials.gov Identifier: NCT04661358).

The main lipidic action of fenofibrate is to lower plasma triglyceride. It also reduces total and low-density lipoprotein (LDL) cholesterol, raises apolipoprotein A1 (apoA1), and high-density lipoprotein cholesterol (HDL), and reduces the concentration of small dense LDL particles and apolipoprotein B. Apart from the increase in apoA1 [32], the other quantitative systemic lipidic actions seem unrelated to its beneficial effects on DR. However, the potential effect of fenofibrate in regulating intraretinal lipid transport [33], and its capacity in modulating the qualitative properties of lipoproteins can contribute to its beneficial effects [34,35].

**Figure 1:**
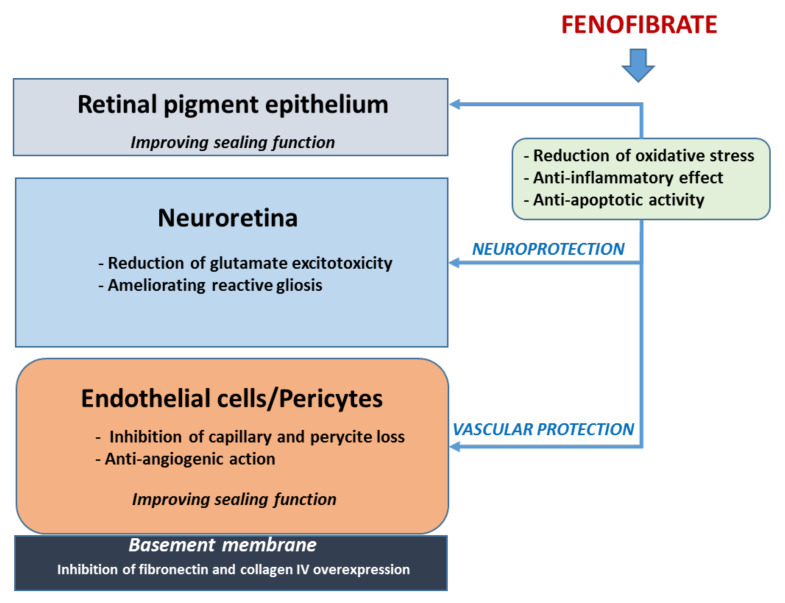
Main non-lipidic mechanisms involved in the beneficial effects of fenofibrate in DR. There are common mechanisms such as the anti-oxidant, anti-inflammatory, and anti-apoptotic actions that take place in the entire retina. However, other actions are only localized in the neuroretina (reduction in glutamate excitotoxicity and reactive gliosis), in vascular cells (inhibition of capillary and pericyte loss), or in the basement membrane (reduction in fibronectin and collagen IV overexpression). As a global consequence, an improvement of sealing function of both outer an inner blood–retinal barrier occurs, thus preventing or arresting the vascular leakage.

Overall, the non-lipidic actions of fenofibrate seem more important in accounting for its effect in reducing the progression of DR than the lipidic-mediated mechanisms. The main underlying mechanisms are summarized in Figure 1 [35,36,37,38,39,40,41,42,43,44,45,46]. Since neurodegeneration and microvascular impairment are two important targets of fenofibrate, it seems reasonable that this drug could be useful in the very early stages of DR. However, specific clinical trials aimed at confirming this concept are needed.

In close relationship with fenofibrate, pemafibrate (a selective PPARα modulator) prevented the retinal pathological neovascularization in oxygen-induced retinopathy in mice. However, further research on the underlying mechanisms is needed [47].

### 3.2. Calcium Dobesilate (CaD)

Two randomized placebo-controlled trials demonstrated the effectiveness of calcium dobesilate (CaD) in preventing the progression of early stages of DR [48,49]. However, another randomized, placebo-controlled study (the CALDIRET study) conducted in 635 type 2 diabetic patients with mild-to-moderate non-proliferative DR (NPDR) presenting at the first visit with microalbuminuria and with a follow-up period of five years, showed that CaD did not reduce the risk of the development of clinically significant diabetic macular edema [50]. The main differences in the characteristics of the patients included in this study, in comparison with the two previously mentioned, were the inclusion of patients with more advanced stages of DR and microalbuminuria as per inclusion criteria. In addition, a lower dose of CaD was used (1.5 g/d vs. 2 g/d). Taken together, these results suggest that CaD is beneficial in the very early stages of DR but its effectiveness in more advanced stages remains to be determined.

CaD has been approved for the treatment of DR in a large number of countries worldwide for many years but it has not been broadly used in clinical practice. The poor understanding of its mechanisms of action has been one of the reasons for this. However, in recent years, experimental evidence has shown that CaD exerts a multifaceted action on neurovascular unit impairment, thus supporting the observed beneficial effects in the early stages of DR [51]. These pleiotropic effects include anti-inflammatory, anti-apoptotic, and antioxidant actions in both neuroretina and microvessels, thus ameliorating the disruption of the blood–retinal barrier (BRB) and the vascular leakage [52,53,54,55]. An important action of CaD is the inhibition of the upregulation of endothelin-1 (ET-1) and its receptors (ETA-R and ETB-R) induced by diabetes [55]. This effect is essential to understand the effectiveness of CaD in experimental models and in early stages of DR, and it is based on the dual effect (microvessels and neural protection) by blocking receptor A and B of the ET-1 (please see the section “Blocking ET-1 receptors”). The molecular mechanisms involved in the inhibitory effect of CaD on ET-1 remain to be elucidated. However, recent evidence suggests that the anti-inflammatory activity of CaD plays an important role [55]. Furthermore, the inhibition of PKC-delta by CaD might also be involved in the downregulation of ET-1 expression [56].

In summary, as occurs with fenofibrate, CaD presents multifaceted pharmacological effects targeting multiple pathogenic pathways involved in NVU dysfunction. However, further research to better understand the mechanisms of action and the clinical outcomes associated with its use is needed. In this regard, a single-blind, multicenter, cluster-randomized trial including a total of 1272 Chinese patients with mild-to-moderate NPDR is ongoing [57]. However, the planned follow-up of only 12 months seems a serious limitation of this study.

## 4. Administration Route When Treating Early Stages of DR

Although fenofibrate and CaD have found to be effective and safe in a few clinical trials, the systemic administration of drugs to block the main pathogenic pathways involved in DR have two main general problems. First, they can hardly reach the retina at pharmacological concentrations. Second, systemic administration could have serious adverse effects and lead to pharmacologic interferences with other drugs used for the treatment of diabetes and its co-morbidities. Furthermore, when the early stages of DR are the therapeutic target, it would be inconceivable to recommend an aggressive treatment, such as frequent intravitreal injections. In addition, gene therapy for DR remains as promising as it was two decades ago. For all these reasons, topical treatment or photobiomodulation have been postulated as new strategies for treating early stages of the disease [9].

The use of eye drops has not been considered an appropriate route to treat retinal diseases because it was assumed that it did not reach the posterior chamber of the eye (i.e., the vitreous and the retina). However, in recent years, several reports have demonstrated the effectiveness of eye drops containing small molecules in treating early stages of DR in experimental models [58], and it seems that they are reaching the retina by the trans-scleral route [59,60,61]. In addition, topical administration of drugs limits their action to the eye and minimizes the associated systemic effects.

A recent systematic review found a few clinical study using topical eye drops of corticosteroid or non-steroidal anti-inflammatory drug (NSAID) reporting favorable effect on diabetic macular edema (DME) [58]. In addition, two studies [29,62] found neuroprotective agents able to arrest further development in patients with pre-existing signs of neurodegeneration and one found such an agent to induce changes in retinal vascular caliber in mild DR. It is reasonable to expect that the improvement of drug delivery by nanotechnology could increase the efficiency and delay the frequency of topical administration. Taken together, topical eye drops open up a new strategy for treating DR, but there is still a need of large randomized clinical trials with longer follow-up periods.

## 5. Treatment Based on Neuroprotection

Diabetes induces the dysregulation of several proteins with neurotrophic activity synthesized by the retina. The knowledge of these pathophysiological mechanisms that take place in the diabetic retina is important for the design of new therapeutic strategies aimed at preventing or arresting the progression of early stages of DR.

### 5.1. Proteins with Neurotrophic and Angiogenic Properties

The retina synthesizes several proteins with neurotrophic activity that are required for the retinal homeostasis. However, when these proteins are overexpressed, apart from neuroprotection, they also have powerful angiogenic activity [63]. Among these proteins, vascular endothelial growth factor (VEGF), insulin growth factor 1 (IGF-1), erythropoietin (Epo), and secretogranin III (Scg3) are the most relevant.

VEGF is required for normal vascular development and maintains the integrity of endothelial cells via antiapoptotic signaling [64,65,66]. However, it is also an essential pathogenic factor of both DME and proliferative DR (PDR). The main source of VEGF in the eye is RPE and glial cells [64]. Apart from its vasculotropic action, VEGF has important neuroprotective properties [67,68]. In this regard, a dose-dependent decrease in ganglion cells has been reported in rats after blocking all VEGF isoforms [69].

IGF-1, mainly produced by endothelial cells, pericytes, and RPE cells, plays a critical role as a survival factor for both microvascular endothelial cells and the neuroretina [70,71,72].

Epo is mainly produced by RPE and its overexpression is an early event in the retina of diabetic patients [73]. Epo is a potent neuroprotective factor [74,75,76], and strikingly high levels have been found in the vitreous fluid of diabetic patients [77]. Apart from neuroprotection, Epo is a potent physiological stimulus for the mobilization of endothelial progenitor cells (EPCs) toward injured retinal sites, thus participating in the remodeling of the damaged tissue [78].

The administration of VEGF, IGF-1, and Epo can be envisaged as rationale treatments because, apart from neuroprotection, they could ameliorate capillary drop-out and vasoregression, which are two important hallmarks of early stages of DR [79,80]. However, all of them could favor neovascularization and, additionally, both IGF-1 and Epo could enhance the angiogenic effects of VEGF, thus favoring the development of PDR [63]. It is therefore difficult to propose these neuroprotective proteins as a reliable strategy for treating early stages of DR.

Secretogranin III (Scg3) belongs to the nine-member chromogranin–secretogranin family that regulate the biogenesis of secretory granules [81,82]. In vivo functional assays confirmed that Scg3 selectively promotes angiogenesis and vascular leakage in diabetic mouse vessels, but not in healthy mice [83,84]. By applying a new technology called comparative ligandomics, Scg3 has been identified as a unique diabetes-selective pro-angiogenic endothelial ligand with preferential binding to diabetic vs. healthy vessels [85]. Of the thousands of endothelial ligands identified by the technology, Scg3 had the highest binding activity ratio to diabetic versus healthy retinal vessels, whereas VEGF binds equally well to both diabetic and healthy retinal vasculatures.

In contrast, VEGF induces angiogenesis and retinal vascular permeability in both diabetic and healthy mice. Therefore, it is not surprising that anti-Scg3 has been proposed as an emerging therapy, and recent studies have shown that Scg-3-neutralizing monoclonal antibodies (mAbs) ameliorate retinal vascular leakage in diabetic mice [82]. The results suggest that anti-Scg3 mAbs represent candidates for clinical development to treat patients with DME. In addition, compared with aflibercept, anti-Scg3 mAb, administered either intravitreally or systemically, blocked pathological angiogenesis with no detectable side effects on healthy vasculatures [84]. However, there are two main scientific gaps that should be filled: (1) the Scg3 receptor has not been identified in the diabetic retina; and (2) neuroprotective action of Scg3 has not been proven, and a potential neurotoxicity associated with ani-Scg3 cannot be ruled out. This could be a limiting factor for clinical development of this promising therapeutic approach.

### 5.2. Peptides/Proteins with Neurotrophic and Anti-Angiogenic or Vasculotropic Properties

PEDF (pigment epithelium-derived factor), mainly synthesized by RPE cells, is a protein with neuroprotective properties and the most important natural inhibitor of angiogenesis, which is downregulated in DR [85,86]. Therefore, the diabetes-induced decrease in PEDF production exerts a pivotal role in two important pathogenic features of DR: neurodegeneration and angiogenesis. Apart from its neurotrophic and neuroprotective properties, PEDF also exerts antioxidant and anti-inflammatory effects and reduces oxidative stress and the production of inflammatory markers in DR models [87]. Moreover, in vitro studies in retinal Müller cells suggest that PEDF decreases glutamate excitotoxicity induced by the diabetic milieu by increasing the expression of glutamine synthetase and by preventing glutamate transporter downregulation [88,89,90]. These multitarget properties place PEDF as a suitable candidate for new therapeutic approaches to treat DR. However, its application is restricted because of its instability and short half-life. In addition, PEDF size may limit its utility as a topical therapeutic agent and, therefore, some synthetic PEDF-derived peptides containing biologically active fragments are needed. In this regard, the topical administration (eye drops) of antiangiogenic PEDF60–77 and neuroprotective PEDF78–121 derivatives reduced neurodegeneration and microvascular leakage in the Ins2(Akita) mouse [91]. Furthermore, eye drop delivery of PEDF-34 promoted ganglion cell survival and axon regeneration after optic nerve crush injury in rats [92]. Gene therapy using AAV-mediated PEDF [93] or the engineered episomal vector pEPito (pEPito-PEDF) [94,95] has also exhibited promising results in experimental in vivo models.

SST (somatostatin) is abundantly produced by the retina, with RPE being the main source [18,96]. Since SST receptors (SSTRs) are also produced in the retina, an autocrine action seems important for the reported activities of SST, which include neuromodulation, vessels stabilization, and the regulation of ion–water transport systems [63,97,98]. A downregulation of SST production occurs in early stages of DR [18] and becomes more pronounced in advanced stages [96,99,100]. Consequently, the beneficial action of SST in preventing fluid accumulation and neovascularization is progressively lost, thus favoring the development of DME and PDR. Therefore, SST replacement can be considered a therapeutic target, not only for preventing the neurodegenerative process, but also for more advanced stages of DR, such as DME and PDR. The promising pilot studies published at the beginning of this century showing the effectiveness of SST analogues administered by intramuscular injections for treating advanced DR [101,102] have not been confirmed in larger studies. The main reasons why the systemic administration of SST analogs failed to arrest the progression of DR were recently reviewed [103]; one of the most important was their inability to cross the BRB. By contrast, topical administration of SST prevented retinal neurodegeneration and neurodysfunction assessed by ERG in streptozotocin (STZ)-induced diabetic rats [59] and in db/db mice [104]. The main mechanisms were: (a) anti-inflammatory action; (b) anti-apoptotic activity, thus preventing the misbalance between pro-apoptotic and survival signaling; and (c) the inhibition of diabetes-induced glutamate–aspartate transporter (GLAST) downregulation (an essential molecule for the clearance of glutamate), thus preventing the glutamate accumulation in the retina induced by diabetes [59,104,105]. This pre-clinical evidence lead to the implementation by the European Consortium for the Early Treatment of Diabetic Retinopathy (EUROCONDOR) of the first phase II-III clinical trial using neuroroprotective drugs for treating early stages of DR in type 2 diabetes. This study showed that SST administered by eye drops was able to arrest the progression of neurodysfunction [29]. However, the EUROCONDOR study did not find any effect of two neuroprotective drugs (brimonidine and SST; administered by eye drops) in preventing or arresting microvascular disease [29]. The short follow-up (2 years), the inclusion of a high proportion of patients with no or very mild microvascular disease, and the excellent metabolic control during follow-up could explain these negative findings of the clinical trial.

Nevertheless, topical treatment with either brimonidine or SST caused retinal arteriolar and venular dilation in those patients with preexisting early DR [106]. Therefore, this study demonstrated, for the first time, that long-term topical neuroprotection-induced retinal vascular changes in patients with diabetes, thus opening up a new strategy for treating early stages of DR.

GLP-1 and GLP-1 receptor agonists (GLP-1RAs) exert neuroprotective effects in both the central and peripheral nervous system [107,108]. GLP-1 is expressed in human retinas, mainly in the ganglion cell layer (GCL), and is downregulated in diabetes. By contrast, no differences in GLP-1R expression were observed in either the RPE or neuroretina between donors with and without diabetes [60]. The neuroprotection of GLP-1RAs in terms of preventing ERG abnormalities and morphological features, such as glial activation and neural apoptosis, by using intravitreal injections of exendin-4 (a GLP-1RA) in rats with STZ-induced diabetes [109,110] and in Goto-Kakizaki rats [111], has been reported. However, as previously mentioned, in the early stages of DR, intravitreous injections are inappropriately invasive. In this regard, the topical administration of GLP-1RAs was found to be able to prevent both neurodysfunction (assessed by ERG) and the retinal neurodegenerative process in a spontaneous model of type 2 diabetes (db/db mouse) [60]. Recently, it has been reported that topical administration of GLP-1 not only prevented but also reverted reactive gliosis and restored the number of neuron cells in db/db mice, thus suggesting a neurogenic effect [112].

The main mechanisms involved in neuroprotection conferred by GLP-1RAs as follows. (1) Anti-inflammatory action by decreasing NF-κB, inflammosome, and key pro-inflammatory factors such as IL-1β and IL-6 [60,112]. (2) Inhibition of the excitotoxicity and neuron death mediated by glutamate accumulation in the extracellular space. This is mainly due to the GLP-1RAs inhibitory effect of the GLAST downregulation induced by diabetes [60,109,110]. (3) Antiapoptotic action. GLP-1RAs prevent the upregulation of proapoptotic markers (i.e., FasL, caspase 8, P53/p-P53, Bax) and the downregulation of survival pathways (Bcl-xL, Bcl-2) induced by diabetes in the neuroretina [60,111,112]. In addition, a significant increase in the ratio p-AKT/AKT and the signaling pathway Akt/GSK3b/β-catenin has been observed, which is essential for the survival of the neurons [60,112]. (4) Antioxidant properties. GLP-1 eye drops attenuate oxidative stress by increasing the protein levels of glutathione reductase, glutathione peroxidase, CuZnSOD, and MnSOD in diabetic retinas, favoring DNA repair and neuron cells proliferation [113]. In addition, intravitreal injections of exendin-4 reduces retinal cell death and ROS generation by upregulating Sirt1 and Sirt3 expressions in the retina of early-stage STZ-diabetic rats [114].

In addition to neuroprotective action, GLP-1RAs also exert microvascular protection by the following mechanisms. (1) By preventing the diabetes-induced downregulation of tight junction proteins (i.e., claudin-5 and occluding) [115]. (2) By downregulating essential mediators of the enhancement of vascular permeability, such as VEGF and placental growth factor (PlGF) via AKT/PKB pathways [60,115]. (3) By inhibiting the overexpression of pro-inflammatory cytokines (i.e., IL-1β, TNF-α), which lead to endothelial damage [60,112]. These effects ameliorate the disruption of the BRB and, perhaps more evidently, lead to the vascular leakage. In conclusion, the topical administration of GLP-1 reverts the impairment of the neurovascular unit by modulating essential pathways involved in the development of DR. These beneficial effects on the neurovascular unit could pave the way for clinical trials addressed to confirm the effectiveness of GLP-1 in early stages of DR.

The semaglutide in subjects with type 2 diabetes (SUSTAIN)-6 clinical trial showed clear beneficial effects of semaglutide (a GLP-1R agonist) in terms of cardiovascular disease, but an unexpectedly higher rate of advanced DR was observed in patients treated with semaglutide in comparison with those treated with placebo. Among the potential explanations of this finding, the rapid lowering of blood glucose levels was the most plausible. In fact, a post-hoc analysis confirmed this hypothesis and concluded that the magnitude of the reduction in HbA1c and the presence of pre-exiting DR were the main factors involved in the risk of DR worsening [116].

Dipeptidyl peptidase IV (DPP-IV) inhibitors. It is well-known that GLP-1 is extremely susceptible to the catalytic activity of the enzyme dipeptidyl peptidase IV (DPP-IV), which rapidly degrades GLP-1, showing a half-life in plasma of 1–2 min [117]. Since GLP-1 is produced by the retina and is downregulated by the diabetic milieu, it is reasonable to hypothesize that the inhibition of DPP-IV could enhance GLP-1, thus preserving its autocrine/paracrine neuroprotective effects. In addition, this strategy could also prevent the degradation of serum-derived GLP-1 that reaches the retina through the blood–retinal barrier. This hypothesis has recently been confirmed in db/db mice treated with eye drops of sitagliptin and saxagliptin (two DPP-IV inhibitors) [118]. The results, in terms of preventing neurodegeneration, were similar to those obtained by using GLP-1RAs and revealed that topical administration of either saxagliptin or sitagliptin induced an increase in the retinal content of GLP-1. In addition, both DPP-IV inhibitors were able to significantly increase the levels of exchange protein activated by cAMP-1 (EPAC-1) [118]. This protein is a downstream cAMP signaling mediator and plays an important role in the maintenance of the endothelial barrier and neuronal functions [119].

DPP-IV inhibitors have several characteristics that might be perceived as advantages over GLP-1RAs when planning a clinical development. (1) Although the main actions of DPP-IV inhibitors seem mediated by an increase in retinal GLP-1 levels, the simultaneous activation of other mechanisms unrelated to GLP-1/GLP-1R cannot be ruled out. These pleiotropic actions are always desirable in a multi-pathway diseases, such as DR. (2) DPP-IV inhibitors are cheaper and more stable than GLP-1. (3) The RPE of subjects with diabetes present higher DPP-IV concentrations than non-diabetic controls matched by age, thus decreasing the availability of GLP-1 for reaching the neuroretina. In fact, the drugs that reach the retina via the trans-scleral route, as is the case for GLP-1, are first challenged by the choroids and the RPE [120]. Therefore, a therapeutic strategy combining the topical administration DPP-IV inhibitors and GLP-1 (topical or administered by the systemic route) could represent a new and more effective approach to treating DR. However, specific studies addressed to confirm this hypothesis are needed.

When a blood glucose-lowering agent is systemically administered and a beneficial effect in the development or progression of DR is observed, it is virtually impossible to know whether this effect is only attributable to the improvement of metabolic control or to its potential direct effect on the retina. Since topical ocular administration of GLP-1 or DPP-IV inhibitors does not influence blood glucose levels, the beneficial effects should be attributed to the direct effect of these drugs on the diabetic retina. This is an important point that supports the use of local administration when designing preclinical proof-of-concept studies for testing any eventual beneficial effect of antidiabetic drugs on the retina independently of their capacity to lower blood glucose levels.

### 5.3. Blocking ET-1

ET-1 and its receptors (ETA-R and ETB-R) are early upregulated in the diabetic retina [53,59,118,119]. The activation of ETA-R mainly mediates vasoconstriction and vasoregression [120], and the activation of ETB-R induces retinal neurodegeneration [121,122,123,124,125]. This dual deleterious effect on both microvasculature and neurons suggests that the ET-1 system plays an essential role in neurovascular unit impairment. In support of this concept, it has been reported that the topical administration of bosentan (a dual endothelin receptor antagonist) was effective in preventing retinal neurodegeneration in db/db mice [59]. Moreover, bosentan inhibited the upregulation of both TNF-α and VEGF, thus preventing the hyperpermeability induced by these cytokines in human retinal endothelial cells. All these findings suggest that therapeutic strategies based on blocking ET-1 should be tested in clinical trials.

The main mechanisms of action of the most representative eye drops for treating early stages of DR are summarized in Table 1.

### 5.4. Neurotransmitters

#### 5.4.1. Proteins Involved in Synaptic Connectivity

**Figure 2:**
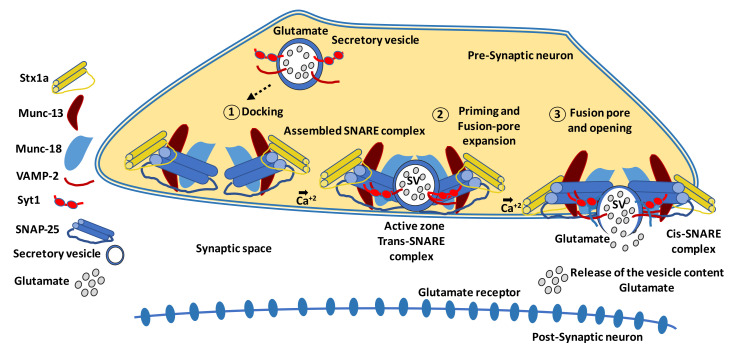
Scheme of an excitatory synapse. Pre-synaptic proteins are organized in a functional complex, playing different roles in vesicular exocytosis. Membrane fusion is a key process for neurotransmitter release. SNARE proteins are the central constituents of the eukaryotic fusion machinery that triggers the fusion of synaptic vesicles (SV), whose membranes contains synaptotagmin 1 (Syt1) and synaptobrevin (VAMP-2), with the plasma membrane [134,135]. The exocytosis stages comprise a docking step, which occurs when the synaptic vesicle comes into contact with the presynaptic plasma membrane named active zone. VAMP-2 then assembles with syntaxin (Stx1a) and SNAP-25 to form a core trans-SNARE complex. Next, the priming step makes the vesicle competent for fusion and fusion pore formation [136]. Finally, the fusion pore opening is followed by fusion pore expansion leading to release of the vesicle contents with the neurotransmitter [137]. Two active-zone proteins, Munc-18 and Munc-13, are key regulators of neurotransmitter release due to their action on the assembly of the SNARE complex, composed by Stx1a, SNAP-25, and VAMP-2 [138,139]. Stages of exocytosis are as follows. (**1**) Docking: the synaptic vesicle and the plasma membrane are brought into contact. (**2**) Priming: this step renders the vesicle as being fusion-competent and enables fusion pore formation. (**3**) Fusion pore opening and release or exchange of the vesicle content.

The neurodegeneration that occurs in the early stages of DR leads to an alteration in the dendritic structure of neurons, resulting in the loss of synaptic connectivity [17,23,127]. In this regard, there is evidence in murine models that diabetes reduces the retinal content of several presynaptic proteins critical to the exocytosis of neurotransmitters and synaptic maintenance [128,129,130]. Vesicular exocytosis mostly involves at least three different steps: (1) docking of a transport vesicle to the plasma membrane; (2) ATP-dependent priming of the vesicle; and (3) fusion of the vesicle to the plasma membrane, which is often activated by extracellular stimuli [131,132,133]. A scheme of this process, including the main proteins involved, is shown in Figure 2 [134,135,136,137,138,139]. In postmortem human retinas, the protein content of synapsin 1 and other neuronal proteins (synaptophysin, SNAP-25, and neurofilament) was depleted compared to non-diabetic donors [140].

Avoiding the downregulation of presynaptic proteins induced by diabetes would be a desirable effect of any neuroprotective drug. In this regard, the diabetes-induced reduction in synaptic proteins was corrected by insulin, but only if the treatment was initiated soon after the onset of diabetes in STZ-induced diabetic Sprague–Dawley rats. By contrast, when insulin was given after a more protracted period of diabetes, the synaptic protein content was not returned to control levels [141]. These findings suggest that the initial phase of synaptic protein loss is reversible and is likely due to the exposition of the retina to the diabetic milieu, whereas the more chronic deficit is permanent and may be due to irreversible cell death. Taurine [142] and lutein [143] may also protect the diabetic retina, improving retinal synaptic connections.

Hyperphosphorylated-tau, a critical toxic mediator in diabetic RGCs synaptic neurodegeneration [144], is downregulated by the topical ocular administration of liraglutide (a GLP-1RA) via activation of GLP-1R/Akt/GSK3β signaling [145]. We have found similar results using topical administration of sitagliptin (a DPP-IV inhibitor) (unpublished results).

In animal models of glaucoma, early synaptic loss and dendritic atrophy are dependent on C1QA, a component of the C1 complex of the complement cascade [146]. Since complement activation occurs in DR [147], it is reasonable to hypothesize that complement-modulating therapeutics could play a role in the prevention of retinal neurodegeneration.

#### 5.4.2. Proteins Involved in Axonal Transport

There is emerging evidence indicating that impairment of axonal transport could be implicated in the neuronal cell death that occurs in neurodegenerative diseases [148]. In fact, in animal models of diabetes, there is evidence indicating that axonal transport is impaired in RGCs [149]. A proteomic analysis of human retinal samples in the early stages of diabetes revealed the upregulation of proteins involved in anterograde transport (i.e., the dynein-dynactin complex) and a downregulation of proteins involved in retrograde transport (i.e., members of the kinesin family) in the retinas from patients with glial activation in comparison with the retinas from diabetic donors without glial activation or non-diabetic donors [150,151]. Therefore, the retinas with glial activation exhibit an imbalance between retrograde and anterograde transport, which could participate in the neurodegenerative process that occurs in DR. In addition, the same proteomic analysis revealed that proteins forming the axon initial segment (AIS) were downregulated in retinas with glial activation [150,151]. The AIS is localized at the proximal part of the axon and separates the somatodendritic domain from the axonal domain, acting as a diffusion barrier between the two neuronal compartments [152,153,154]. The AIS is essential for the initiation of the axon potential, axonal regeneration, and the maintenance of axonal polarity, and has been implicated in other neurodegenerative diseases, such as Alzheimer’s disease [152].

It should be emphasized that proteins involved in synaptic connectivity and axonal transport are essential for neuronal survival. Therefore, therapeutic strategies addressed to avoid the imbalance that occurs in DR are foreseeable, exerting beneficial effects on NVU impairment. However, further research in this field is needed.

## 6. Treatment Based on Targeting Inflammation

### 6.1. General Considerations

Inflammation plays a major role in the pathogenesis of NVU impairment and, therefore, could be a key therapeutic target in the early stages of DR [9,155,156,157]. Risk factors of DR, such as hyperglycemia, hypertension, and dyslipidemia, are all involved in retinal inflammation in DR through a variety of mechanisms including oxidative stress, NOS dysregulation, AGE formation, RAS activation, and the inhibition of endogenous anti-inflammatory pathways [158].

Among the proinflammatory cytokines/chemokines involved in the pathogenesis of DR, the IL-1β, TNF-α, monocyte chemotactic protein-1 (MCP-1), stromal cell-derived factor-1 (SDF-1), and the adhesion molecules (ICAM-1, VCAM-1, sVAP-1) play a relevant role [159,160,161,162]. The main source of proinflammatory cytokines in DR is local synthesis by the retina [9], and several studies have pointed out retinal Müller glial cells and microglia as the initiators of retinal neuroinflammation [159]. Additionally, RPE cells also secrete proinflammatory cytokines, such as TNF-α, IL-6, and IL-8 [163]. Furthermore, activated monocytes in the extravascular space differentiate into macrophages that also secrete pro-inflammatory cytokines [164]. The increased level of inflammatory mediators leads to an early, persistent chronic inflammatory condition in the diabetic retina, resulting in leukocyte activation, adhesion to the vascular endothelium (leukostasis), and disruption of the BRB [165,166], the main cause of DME.

As commented above, Müller cells (macroglia) and microgial cells play a key role in the inflammatory process that occurs in the setting of diabetes-induced retinal neurodegeneration. Following retinal stress, the expression of vimentin and GFAP are dramatically upregulated in Müller cells leading to the stiffness of the glial cells, which exerts an inhibitory effect on both neural [167,168] and capillary regeneration [169]. In addition, Müller cells are living optical fibres that guide light through the inner retinal tissue to photoreceptors [170]. They thereby enhance the signal–noise ratio, by minimizing intraretinal light scattering, and conserve the spatial distribution of light patterns in the propagating image [171]. It was shown that Müller cells transmit predominantly red-green and less violet-blue light [172], and recent research has indicated that Müller cells support high acuity vision predominant by the guidance of red-green light to M-opsin-expressing cones [173]. All of these findings suggest that reactive gliosis could contribute to early visual abnormalities detected in subjects with diabetes.

Retinal microglia consist of resident immune cells localized in the inner and outer plexiform layers of the retina, which are activated in several retinal degenerative and inflammatory diseases [174,175]. When activated their appearance changes from a highly ramified to an amoeboid morphology [175]. Neurons, by releasing chemokines in response to injury, play an important role in activating microglia [176,177,178].

Activated Müller cells and microglia not only trigger a deleterious inflammatory process, but also serve as rapid sensors of neuronal damage and are responsible for tissue repair and neural regeneration through various mechanisms [179,180,181,182]. Thus, the release of proinflammatory cytokines by activated Müller cells and microglia is a double-edged sword because, depending on the intensity, they could protect photoreceptors and neurons from cell death, but also induce the breakdown of the BRB and neural death. This dual action should be taken into account in the design of therapeutic approaches addressed to abrogate glial activation.

### 6.2. Targeting Pro-Inflammatory Cytokines

IL-1β is a pivotal inflammatory cytokine because it is able to activate NF-kb, which in turn governs the production of IL-8, MCP-1, and TNF-α [183]. IL-1β induces pericyte apoptosis via NF-κB activation and increases endothelial permeability in DR [184]. Moreover, IL-1β plays a relevant role in the degeneration of retinal capillaries [185] and is able to downregulate the interphotoreceptor retinoid-binding protein (IRBP), an essential protein for the maintenance of photoreceptors [19]. Therefore, limiting IL-1β-triggered the inflammatory processes, and blocking its receptor could represent valid approach for treating DR. In this regard, it has been shown in rodents that the systemic administration of antioxidants [180], minocyclyne [185], or topical (eye drops) of SOCS-1 [126], SST [104], GLP-1 [60], and DPP-IV inhibitors [118] inhibit the upregulation of IL-1β induced by diabetes. Furthermore, neutralization of IL-1β by the administration of IL-1ab increased glutamine synthetase and glutamate–aspartate transporter (GLAST) expression in retinal Müller cells under hypoxia, thus reducing glutamate excitotoxicity [186].

TNF-α downregulates tight junction proteins of endothelial cells and is also required for VEGF-induced leakage, thus participating in the breakdown of the BRB [187]. In this regard, it has been reported that TNF-α alone induces small-molecule permeability of the BRB in vitro, whereas the combination of TNF-α, IL-1β, and VEGF induces permeability to large molecules [188]. The intravitreal administration of TNF-a causes retinal ganglion cell death and optic nerve degeneration [189,190]. Moreover, mice genetically deficient in TNF-α were reported to have less diabetes-induced vascular permeability and leukostasis [191]. The systemic administration of eternacept, a soluble TNF-α receptor that acts as a competitive inhibitor to block the effects of TNF-α binding to cells, reduced leukostasis [192], BRB breakdown, and NF-kB activation in the diabetic retina of experimental models [193]. Intravitreal injection of another TNF-α-specific inhibitor, pegsunercept, led to a significant reduction in pericyte loss and capillary degeneration in diabetic rats [194,195]. On the other hand, TNF participates in excitotoxicity, neuronal death, and synaptic dysfunction [196]. However, TNF has also several physiological neuroprotective functions. In the brain, TNF produced by neurons and glial cells acts as an essential gliotransmitter and regulates synaptic communication between neurons, as is demonstrated by its involvement in synaptic scaling and plasticity [197]. In addition, it participates in the learning and memory processes via hippocampal neuronal development [198,199]. Furthermore, TNF also protects against excitotoxicity via TNFR2 [200,201]. Therefore, this dual effect should be taken into account in therapeutic strategies based on long-term TNF-α blockade.

Suppressors of cytokine signaling (SOCS) proteins are a family of intracellular cytokine-inducible proteins, consisting of eight members, and SOCS1 is one of the best characterized [202]. SOCS1 ameliorates cytokine receptor signaling through means of its capacity to inhibit all four JAK kinases [203]. SOCS1 is also a regulator of M1/M2 macrophage functions [202,203] and a key negative regulator of IL-6 and TNF-α. It should be noted that topical administration of SOCS1-derived peptide arrests retinal neuroinflammation and vascular leakage in db/db mice [126].

Apart from targeting pro-inflammatory cytokines, the administration of general anti-inflammatory treatments, such as corticosteroids or non-steroid anti-inflammatory drugs (NEAIDs) by topical route, have been useful for treating DME [204,205] in humans. However, a lack of effect in reducing retinal thickness after 1 year of the topical administration of nepafenac has also been reported in patients with noncentral-involved DME [206]. The systemic administration of aspirin and salicilates, and the inhibition of several mediators involved in the inflammatory response, such as COX-2 and NFkB, have proved useful in reducing the retinal microvascular abnormilities induced by diabetes in experimental models [157]. However, clinical evidence for the effectiveness of anti-inflammatory treatment either through eye drops or systemic administration is lacking and, therefore, further research on this issue is required.

## 7. Concluding Remarks and New Perspectives in Clinical Practice

Neurovascular unit impairment is a main therapeutic target in the early stages of DR. Although our knowledge of the intricate relationship between diabetes-induced retinal neurodegeneration and microvascular disease is still limited, it seems that neurodegeneration is not always the apparent primary event in pathogenesis of DR [14]. In addition, it has recently been shown that the structural and functional deficits appear to be only weakly associated, suggesting that mechanisms in addition to retinal thinning underlie the functional defects in early-stage DR [207]. This new comprehensive understanding of the natural story of DR entails the necessity of a better phenotyping and stratification of patients with DR. Nevertheless, neuroprotection itself is important to prevent or arrest deficiencies in sensory capacity, including decreased hue discrimination, contrast sensitivity, delayed dark adaptation, and abnormal visual fields, which result in reduced vision-related quality of life [208,209,210]. In addition, the assessment of neurodegeneration or neurodysfunction is now emerging as an essential determinant, besides microvascular impairment, of the final visual outcome of patients with diabetes [5].

The gold standard methods to identify the presence of neurodegeneration are multifocal electroretinography (mfERG) and frequency domain optical coherence tomography (FD-OCT) [8]. These methods permit us to detect functional and morphological abnormalities, respectively, even before microvascular abnormality can be observed under ophthalmoscopic examination. The functional assessment of neurodegeneration by mfERG is a cumbersome and time-consuming examination and, for this reason, it is generally reserved for clinical trials. In recent years, microperimetry and the flicker ERG handheld recording device (RETeval) have emerged as promising methods to be used in clinical practice due to their simplicity and acceptable level of accuracy [211,212,213]. Another device, the dynamic vessel analyzer (DVA) makes it possible to detect NVU impairment by measuring flicker light-induced changes in the diameter of retinal arterioles [214,215]. It has been reported that retinal vessel responses to flicker light are diminished in subjects with DR, and decrease progressively with increasing DR severity [216,217]. In addition, reduced retinal arteriolar and venular dilatory responses to flickering light are associated with the risk of DR progression at 1 year in adult patients with diabetes [218]. Regarding structural assessment, angio-OCT has proved to be a very useful tool for identifying microvascular alterations in early stages of DR [219]. Therefore, all these methods seem appropriate for monitoring the effects of new treatment approaches in the early stages of DR. However, standardization and cost-effectiveness studies are still required before their widespread use in clinical practice can be recommended.

In the coming years, it is foreseeable that a position statement will be made by the scientific community in terms of selecting the most cost-efficient examinations that permit us to identify a phenotype of diabetic patients in which neurodegeneration has a key role in the development of DR and, therefore, those patients in whom neuroprotection should be more effective. Nevertheless, taking into account that several therapeutic approaches (i.e., GLP-1Ras, DPP-IV inhibitors, blocking ET-1) have shown a dual action (neuroprotective and vasculotropic) in experimental studies, it seems reasonable to promote this type of holistic approach in treating early stages of DR rather than in exclusively neuroprotective agents. The possibility of using topical therapy for this purpose opens up a new and safe strategy which should be tested in appropriate clinical trials.

Another critical point is to change the metrics used in the assessment of clinical trials based on the current regulatory framework (ex. 2–3 steps of progression in the ETDRS scale). The new methods of retinal assessment above mentioned could revolutionize the design of clinical trials aimed at testing new drugs for DR treatment in terms of a significant reduction in the needed sample size and in shortening the required follow up.

In conclusion, a new scenario is appearing for treating early stages of DR, one of which is based on the better definition of the degree of neurodegeneration and vascular impairment that occurs in an individualized patient. This will allow us to recommend a better targeted and more efficient therapeutic approach in the early stages of the disease.

## Figures and Tables

**Table 1:** Main mechanisms of action of the most representative topical treatments (eye drops) for early stages of DR in experimental models.

Treatment	Reference	Neuro- degeneration	Micro- angiopathy	Mechanisms of Action			
				Glial Inflammation	Neuronal Apoptosis	Oxidative Stress	Vascular Permeability
PEDF	[91]	yes	yes	+	+		+
SST	[59,60,61,62,63,64,65,66,67,68,69,70,71,72,73,74,75,76,77,78,79,80,81,82,83,84,85,86,87,88,89,90,91,92,93,94,95,96,97,98,99,100,101,102,103,104]	yes		+	+		
GLP-1	[60,112, 113]	yes	yes	+	+	+	+
DPP-IVi	[118]	yes	yes	+	+	+	+
Bosentan	[61]	yes	yes	+	+		+
SOCS-1	[126]	yes	yes	+	+		+

PEDF: pigment epithelium-derived factor; SST: somatostatin; GLP-1: glucagon-like derived peptide 1; DPP-IVi: dipeptidyl peptidase IV inhibitors; SOCS-1: suppressors of cytokine signaling-1.
